# Altered thalamic microstructure in migraine without aura patients: a diffusion tensor magnetic resonance imaging study

**DOI:** 10.1186/1129-2377-14-S1-P121

**Published:** 2013-02-21

**Authors:** G Coppola, E Tinelli, C Lepre, E Iacovelli, C Di Lorenzo, G Di Lorenzo, F Pauri, G Fiermonte, F Bianco, F Pierelli

**Affiliations:** 1 Sapienza University of Rome Polo Pontino, Department of Medico-Surgical Sciences and Biotechnologies, Latina, Italy; 2 Sapienza University of Rome, Department of medico-surgical sciences and biotechnologies, Neurology section, Rome, Italy; 3 Tor Vergata University of Rome, Psychiatric Clinic, Department of Neuroscience, Rome, Italy

## Background and objectives

Studies of spontaneous EEG and visual or somatosensory evoked high frequency oscillations indicate that the abnormal fluctuations of cortical responsivity over time in relation to the migraine attack could be due to abnormal thalamic control. Here we searched for possible structural changes in the thalamus of migraineurs by mean of acquiring diffusion tensor magnetic resonance imaging (MRI). This MRI technique provides quantitative data on water molecular motion, as a marker of tissue structure. Materials & Method – Seventeen untreated migraine without aura (MO) patients underwent MRI scan (3-Tesla Siemens Gyroscan) during (n=7) and between attacks (n=10) and were compared to a group of 14 healthy volunteers (HV). We examined fractional anisotropy (FA) and mean diffusivity (MD) in the thalamus.

## Results

Between attacks MO patients had a significantly higher FA and lower MD values in the bilateral thalami when compared to HV (p<0.05). During attacks, all MRI quantitative measurements in migraineurs were similar to those found in HV. In MO patients, FA of the right thalamus was positively correlated with the number of days since the last migraine attack (r=0.588, p=0.034).

## Conclusion

The higher thalamic FA values noted between attacks in MO patients may be related to a decrease in regional branching and crossing of fibers, which normalizes during an attack. Whether these changes could be considered as the anatomical counterpart of the cyclic functional fluctuations previously observed with the neurophysiology in migraine remains to be determined.

